# A composite improved attention convolutional network for motor imagery EEG classification

**DOI:** 10.3389/fnins.2025.1543508

**Published:** 2025-02-05

**Authors:** Wenzhe Liao, Zipeng Miao, Shuaibo Liang, Linyan Zhang, Chen Li

**Affiliations:** ^1^School of Artificial Intelligence, Hebei University of Technology, Tianjin, China; ^2^Tianjin Key Laboratory of Environment, Nutrition and Public Health, Department of Occupational and Environmental Health, School of Public Health, Tianjin Medical University, Tianjin, China

**Keywords:** electroencephalography, convolution neural network, attention mechanism, temporal convolution network, motor imagery, classification

## Abstract

**Introduction:**

A brain-computer interface (BCI) is an emerging technology that aims to establish a direct communication pathway between the human brain and external devices. Motor imagery electroencephalography (MI-EEG) signals are analyzed to infer users’ intentions during motor imagery. These signals hold potential for applications in rehabilitation training and device control. However, the classification accuracy of MI-EEG signals remains a key challenge for the development of BCI technology.

**Methods:**

This paper proposes a composite improved attention convolutional network (CIACNet) for MI-EEG signals classification. CIACNet utilizes a dual-branch convolutional neural network (CNN) to extract rich temporal features, an improved convolutional block attention module (CBAM) to enhance feature extraction, temporal convolutional network (TCN) to capture advanced temporal features, and multi-level feature concatenation for more comprehensive feature representation.

**Results:**

The CIACNet model performs well on both the BCI IV-2a and BCI IV-2b datasets, achieving accuracies of 85.15 and 90.05%, respectively, with a kappa score of 0.80 on both datasets. These results indicate that the CIACNet model’s classification performance exceeds that of four other comparative models.

**Conclusion:**

Experimental results demonstrate that the proposed CIACNet model has strong classification capabilities and low time cost. Removing one or more blocks results in a decline in the overall performance of the model, indicating that each block within the model makes a significant contribution to its overall effectiveness. These results demonstrate the ability of the CIACNet model to reduce time costs and improve performance in motor imagery brain-computer interface (MI-BCI) systems, while also highlighting its practical applicability.

## 1 Introduction

Brain-computer interface (BCI) has ushered in a new era of human-technology interaction. It establish a direct communication bridge between brain neural signals and external devices (Wolpaw et al., 2002). BCI systems can accurately measure and interpret brain activity, translating an individual’s intentions, thoughts, or perceptions into executable control signals. These signals can operate various external devices, including wheelchairs, prostheses, computer cursors, and advanced robotic systems. This allows users to perform specific tasks (Do et al., 2013; Ramadan and Vasilakos, 2017; Vilela and Hochberg, 2020).

Electroencephalography (EEG) is a non-invasive technique for capturing electrical signals generated by neuronal activity in the brain. These signals are detected by electrodes placed on the scalp. The electrodes can detect small current fluctuations generated by neuronal populations in the cerebral cortex. Due to its high temporal resolution and portability, EEG is not only an indispensable tool in clinical medicine but also widely used in engineering and psychology (Biasiucci et al., 2019).

Motor imagery (MI) refers to the mental rehearsal of movements without actual physical execution (Pfurtscheller and Neuper, 2001). This process includes recalling past actions and imagining future ones. Monitoring sensory motor rhythms (SMR) shows that MI induces event-related synchronization (ERS) and event-related desynchronization (ERD). This marks it as an actively evoked EEG signal (Grandchamp and Delorme, 2011). Motor imagery electroencephalography (MI-EEG) signals are widely used in rehabilitation medicine to support the recovery of compromised motor functions. A key advantage of these signals is that they can autonomously activate motor-related brain regions without relying on external stimuli. Motor imagery brain-computer interface (MI-BCI) systems have been applied across a range of medical and non-medical fields. In medicine, MI-BCI applications include stroke rehabilitation, prosthetic control, wheelchair navigation, psychological therapy, and cognitive training (Khan et al., 2020). Beyond medical applications, MI-BCI systems are used in vehicle and drone control, gaming, skill development, and virtual reality.

Despite the significant potential of MI-BCI across various domains, challenges persist in accurately interpreting users’ intentions. EEG patterns generated during MI can vary substantially between subjects, increasing the time and complexity required for system customization. This limits the universal applicability of MI-BCI technology. The non-stationarity of MI-EEG signals is a primary obstacle in MI-BCI development. Variations in user fatigue and attention levels further exacerbate signal non-stationarity (Abdulkader et al., 2015). Motion artifacts, such as electromyographic activity, eye movements, and blinking-related electrooculogram (EOG) signals, can significantly degrade EEG signal quality (Rashmi and Shantala, 2022). This ultimately impacts the performance of MI-BCI systems. Consequently, inter-individual variability and signal non-stationarity present substantial challenges for the practical implementation of MI-BCI. These challenges make it important to classify MI-EEG signals accurately.

Researchers have proposed various methods for capturing MI-EEG signals features and classifying MI tasks. Conventional feature extraction methods typically involve fewer parameters and have lower computational complexity. These methods include common spatial patterns (CSP) (Ramoser et al., 2000), fourier transform (FT) (Wang P. et al., 2018), and wavelet transform (WT) (Xu et al., 2018). CSP is a typical feature extraction algorithm. It is capable of extracting the spatial distribution components of each class from multi-channel EEG signals. It is suitable for processing small datasets and EEG signals with minimal motion artifacts. In MI-EEG signal classification, support vector machine (SVM) (Chatterjee and Bandyopadhyay, 2016) is the most representative method. SVM perform well in classification tasks with small sample sizes and well-defined features. However, its performance drops when handling large datasets. Ang et al. (2012) proposed filter bank common spatial pattern (FBCSP). This method combines a set of band-pass filters with CSP. The accuracy obtained by combining this method with SVM shows a slight improvement over CSP, but due to the high noise level in EEG signals, the results remain unsatisfactory. Traditional approaches to MI-EEG feature extraction have limited efficacy in non-linear models, especially in complex or multi-class classification scenarios. Their performance is significantly reduced in these cases.

In recent years, the use of deep learning (DL) techniques for classifying MI-EEG signals has increased rapidly. DL approaches can automatically extract features from data and possess strong nonlinear fitting capabilities. They introduce nonlinear factors through activation functions, making them effective in handling the complex nonlinear characteristics of EEG signals. This has led to significant improvements in the performance of MI-EEG signal classification. In recent years, many DL architectures have been introduced for MI task classification, including convolutional neural network (CNN), recurrent neural network (RNN), long short-term memory (LSTM), and autoencoder. Additionally, various CNN modifications have been proposed, such as multi-branch CNNs, multi-scale CNNs, and Residual-based CNNs. Lawhern et al. (2018) proposed EEGNet, a compact CNN architecture designed for EEG data, which has been effectively applied in various BCI paradigms. Its lightweight design makes EEGNet particularly suitable for EEG analysis. Chen W. et al. (2024) proposed the EEGNeX model, which replaces the two-dimensional (2D) convolutions and separable convolutions in EEGNet with a pair of 2D convolutions. Luo et al. (2018) developed an RNN model that incorporates a sliding window cropping strategy (SWCS) for classifying MI-EEG signals. Wang Z. et al. (2018) proposed a classification framework that utilizes LSTM and uses a one-dimensional aggregation approximation (1d-AX) technique to extract effective signal representations. In the field of algorithmic integration, Lu et al. (2019) combined one-dimensional CNN with LSTM for classifying MI tasks. Later, Gao et al. (2022) integrated gated recurrent units (GRU) with CNN to create a parallel feature fusion network. Overall, CNN models have found broader application in MI task classification than other DL models. They have also proven to be compatible with integration into other DL models.

Temporal convolutional network (TCN) has been increasingly applied in various fields (Bai et al., 2018). TCN is particularly useful in time series forecasting and sequential annotation tasks. It effectively extracts both high-frequency and low-frequency information from sequences. TCN is a specialized one-dimensional CNN composed of three main components: causal convolution, dilated convolution, and residual blocks. Causal convolution ensures strict temporal constraints, while dilated convolution enhances the network’s ability to process long sequences (Bi et al., 2021). Unlike conventional RNN, TCN uses convolutional operations to capture temporal interdependencies. This effectively expands the receptive field and supports long-range dependencies (Hewage et al., 2020). Recent research has applied the TCN framework to classify MI-EEG signals. Ingolfsson et al. (2020) proposed the EEG-TCNet model. It combines the EEGNet and TCN architectures. Subsequently, Musallam et al. (2021) proposed an enhanced version of EEG-TCNet through feature concatenation, named TCNet-Fusion. Salami et al. (2022) proposed the EEG-ITNet model. It is a tri-branch structure combining CNN and TCN to extract rich temporal and spatial information.

Attention is the cognitive ability to selectively focus on specific objects. Drawing inspiration from this capacity, researchers have introduced the attention mechanism. In deep learning, the attention mechanism dynamically adjusts the model’s focus across different segments of input data. This enhances the model’s performance and efficiency by emphasizing more significant information. In 2017, Google researchers introduced the Transformer architecture, a novel neural network framework based entirely on attention mechanisms. Later, Hu et al. (2018) proposed the squeeze-and-excitation (SE) model. It focuses on inter-channel relationships and enables the model to autonomously determine the significance of different channel features. In the same year, Woo et al. (2018) proposed the convolutional block attention module (CBAM), an attention mechanism that retains conventional channel attention and adds a spatial attention mechanism. It enhances network performance across both channel and spatial domains. Recently, the scientific community has increasingly adopted DL models with attention mechanisms for classifying MI-EEG signals. Song et al. (2022) combined CNN with self-attention to classify tasks involving MI and emotion recognition. Altaheri et al. (2022) proposed ATCNet, a model that integrates multi-head self-attention (MSA), TCN, and CNN to decode MI-EEG signals. Hu et al. (2023) proposed MSATNet, a model that combines a dual-branch CNN and Transformer to classify MI-EEG signals. Xie et al. (2024) proposed the BFATCNet model, designing a tri-branch structure with attention mechanisms and TCN. Similarly, Deng et al. (2024) developed a tri-branch architecture that combines parallel multi-head attention with SE and CBAM alongside EEGNet and TCN for MI-EEG signal classification.

This study proposes a novel composite improved attention convolutional network, CIACNet, for MI-EEG signals classification. The proposed CIACNet model utilizes a dual-branch convolutional architecture, combining an improved CBAM with TCN for feature extraction from MI-EEG signals. The structure of this model differs from the repetitive multi-branch structures used by others, as it employs multi-level feature concatenation, allowing the model to consider the features obtained from each block component. This lightweight model removes the requirement for human intervention in the decoding process. Its applicability within MI-BCI systems is highlighted by its automated decoding, high accuracy, and reduced training costs for subjects. The steps for decoding within the proposed CIACNet model are as follows: First, conduct simple preprocessing. Second, extract rich temporal features using a dual-branch CNN with distinct parameter sets. Third, apply the improved CBAM to enhance the model’s ability to extract key information. Fourth, capture high-level temporal features using the TCN. Finally, in the fully connected (FC) layer, we concatenate the features from multiple branches and perform classification using the softmax function. This research highlights the following contributions:

1. We have proposed the high-performance CIACNet model, which combines CNN, improved CBAM, and TCN to form a composite deep learning architecture. The model has demonstrated exceptional performance on the BCI Competition IV-2a and BCI Competition IV-2b datasets.
2. The dual-branch CNN architecture effectively extracts features from MI-EEG signals at multiple scales, thereby enhancing the comprehensiveness of the feature learning process.
3. The improved CBAM further enhances the model’s ability to extract key information by modifying the pooling approach of the standard CBAM. This attention mechanism effectively identifies the importance of different regions within the input feature map across both channel and spatial dimensions.
4. In the FC layer, concatenating features from different levels enhances the model’s ability to represent complex features. This improves the overall classification accuracy.

## 2 Methods

**Figure 1:**
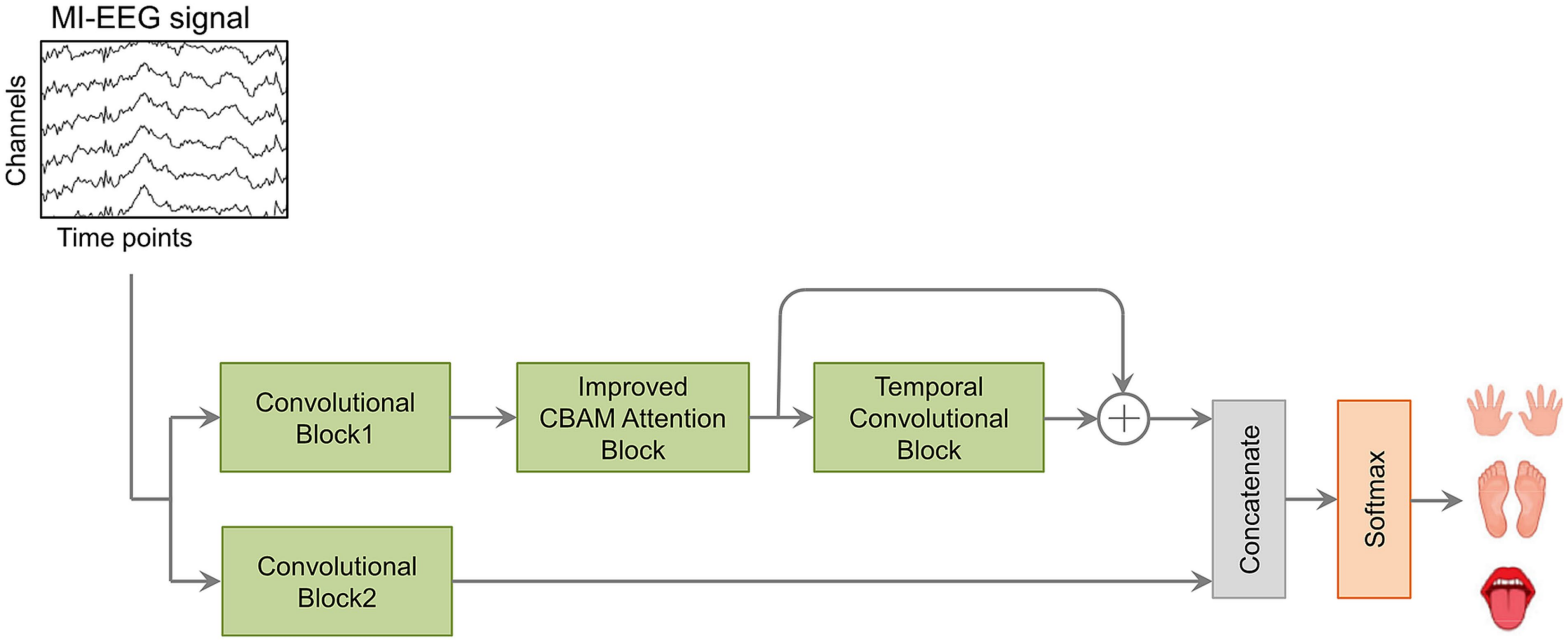
The components of the CIACNet model.

The proposed CIACNet model consists of three core components: the convolutional (CV) block, the improved CBAM attention (IAT) block, and the temporal convolution (TC) block, as shown in Figure 1. The CV block includes two subblocks, CV1 and CV2. Each subblock comprises three distinct convolutional layers: temporal, channel depth-wise, and spatial convolutions. This block is responsible for encoding the spatiotemporal features of MI-EEG signals. The CV block captures temporal features across different time steps and outputs a comprehensive representation of the signals. The IAT block is equipped with an improved CBAM. It effectively captures features from both channel and spatial perspectives. The TC block utilizes TCN to extract high-level temporal features from sequential data. In the final stage of the model, the outputs from the IAT, TC, and CV2 blocks are concatenated. The resulting features are then passed to the FC layer with a softmax classifier. Further details about the CIACNet model will be provided in the subsequent sections.

### 2.1 Data preprocessing and input representation

In the proposed CIACNet model, the raw MI-EEG signals undergo a simple preprocessing phase. During this phase, to preserve the original characteristics of EEG signals and reduce the latency of the decoding system, we utilize the full spectrum of EEG frequencies and all channels without removing any artifacts. A single trial dataset of MI-EEG can be denoted as $X_{i}\in {\mathbb{R}}^{C\times T}$ , where the corresponding class label is $y_{i}\in \left\{1,2,\ldots ,N\right\}$ . C , T , and N represent the channel count, the number of sampling points, and the class count, respectively. To address the non-stationarity of the signals, we apply Z-score normalization, as shown in Equation 1.


(1) $${X_{i}}^{\prime }=\frac{X_{i}-\mu }{\sqrt{\sigma^{2}}}$$

Where $X_{i}$ and ${X_{i}}^{\prime }\in {\mathbb{R}}^{C\times T}$ denote the input and normalized dataset, respectively. μ and $\sigma^{2}$ represent the mean and variance of the training dataset.

The normalization process ensures that each channel in the raw EEG signals has a mean of zero and a standard deviation of one. MI-EEG datasets usually contain multiple trials conducted by different subjects. For a given subject, the MI-EEG data is represented as a collection $A_{n}=\left\{\left(X_{1},y_{1}\right),\left(X_{2},y_{2}\right),\ldots ,\left(X_{i},y_{i}\right)\right\}$ , where n and i indicate the n-th subject and the i-th trial, respectively. The goal of the CIACNet model is to decode each input trial $X_{i}$ , determine its predicted category, and compare it to the actual class label $y_{i}$ .

### 2.2 Convolutional (CV) block

Drawing inspiration from the EEGNet model, we implemented a series of improvements and introduced the CV1 and CV2 blocks. EEGNet is a compact CNN that demonstrates strong classification capabilities. Its simple architecture limits its effectiveness in processing noisy data. To improve the comprehensiveness of MI-EEG signal decoding, this study incorporates the enhanced CV1 and CV2 blocks as branches within the CV block. The CV block adopts a multi-branch, multi-scale convolutional strategy to enrich the extracted features of the EEG signals.

The CV1 and CV2 blocks serve as dual branches for feature extraction within the model, each receiving the same input. They follow EEGNet’s structure but with different parameter settings. While the original EEGNet employs 2D, depthwise, and separable convolutional layers, we have replaced its separable convolutions with 2D convolutions. This adjustment enhances the model’s ability to learn features and enables it to capture more complex spatial and temporal feature relationships.

**Figure 2:**
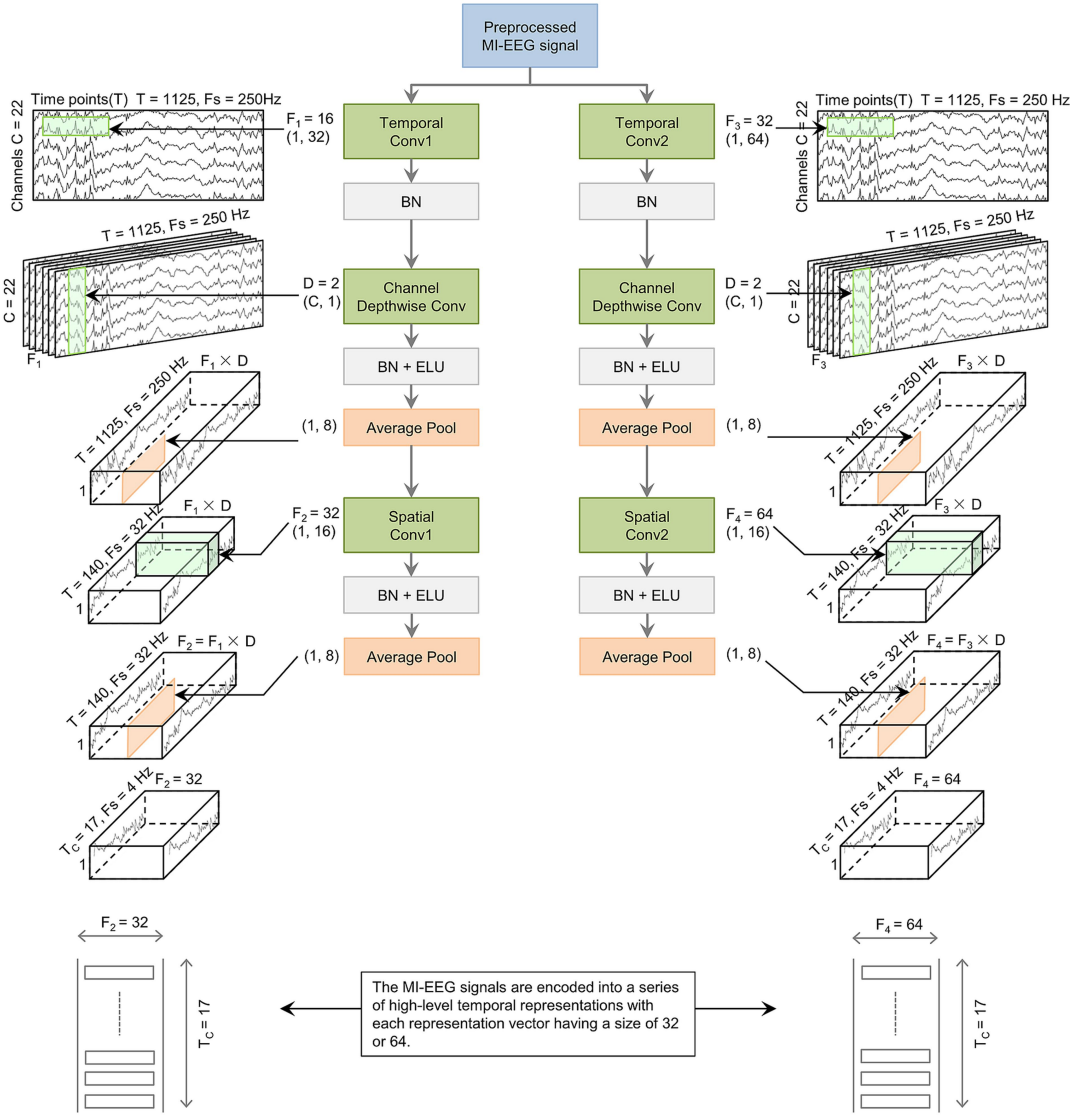
The basic structure of the CV block. The preprocessed MI-EEG data is encoded by the CV block. This encoding outputs a sequence with T_C_ elements. Each element is a vector of size F_2_ or F_4_.

The CV block consists of three convolutional layers, as shown in Figure 2. The data in the figure is based on the BCI IV-2a dataset. The first layer uses 2D convolution, where the CV1 and CV2 blocks apply $F_{1}=16$ and $F_{3}=32$ filters, respectively, for temporal convolution. These filters have dimensions of $\left(1,32\right)$ and $\left(1,64\right)$ based on $K_{C1}=32$ and $K_{C3}=64$ . The CV2 block uses larger filters to cover longer time windows, and a greater number of filters to extract more diverse features. After the first layer, EEG signals produce temporal feature maps. The second layer uses depthwise convolution with $F_{1}\times D$ and $F_{3}\times D$ filters for CV1 and CV2, respectively, each with size $\left(C,1\right)$ , where C represents the number of MI-EEG channels. Based on empirical findings, D is set to 2. Following depthwise convolution, an average pooling layer with dimensions (1, 8) reduces the sampling frequency and temporal sequence length to 32 Hz and 140, respectively. The third layer applies 2D convolution similar to the first layer. In this layer, the CV1 and CV2 blocks use filters of size $\left(1,16\right)$ , with $F_{2}=32$ and $F_{4}=64$ filters, respectively. This is followed by an average pooling layer with size $\left(1,8\right)$ , which reduces the sampling frequency and temporal sequence length to 4 Hz and 17, respectively. After each convolutional layer in the CV block, batch normalization (BN) is applied to standardize the input and accelerate the training process. Additionally, ridge regularization is incorporated into each convolutional layer to control the weight magnitude. Following the second and third convolutional layers, exponential linear unit (ELU) activation functions are utilized to provide non-linear mapping, enhancing network performance. Dropout layers are also included to mitigate overfitting by randomly deselecting neurons. The CV block uses varying filter sizes and quantities across its dual branches. This approach effectively extracts rich features from different scales. As a result, it significantly improves classification accuracy.

### 2.3 Improved CBAM attention (IAT) block

Inspired by the human ability to selectively focus on specific information, attention mechanisms in deep learning have proven effective at decoding MI-EEG signals. CBAM integrates both channel and spatial attention. It is a highly effective attention mechanism (Altuwaijri and Muhammad, 2022). CBAM allows the model to learn the significance of different regions within the input feature map across both channel and spatial dimensions. Notably, CBAM is efficient and lightweight. It imposes minimal additional computational burden on the CNN architecture. The channel attention component of CBAM applies global average and max pooling to the input feature map, generating distinct channel-wise representations. The spatial attention component performs average and max pooling for each channel in the feature map, producing two separate channel feature maps. These maps are then concatenated to form a comprehensive spatial attention map.

Max pooling focuses solely on the largest element and disregards the others within the pooling region. This helps preserve the most significant features but may overlook some valuable information. Average pooling computes the mean value of all elements within the pooling region, which makes it well-suited for output smoothing. In this work, we improve CBAM by incorporating stochastic pooling (Zeiler and Fergus, 2013). This method assigns probabilities to feature map elements based on their numerical values. The likelihood of an element’s selection is proportional to its magnitude. As a result, the pooled features neither overemphasize local maxima nor are dominated by mean smoothing effects. By combining these three pooling strategies, the model is able to extract features more effectively. It can also assess the significance of various regions within the input feature map. This ultimately improves the performance of the attention block.

Instead of using deterministic selection methods, stochastic pooling adopts a probabilistic approach to select elements. It randomly selects elements based on a probability distribution derived from the activations within the pooling region. The likelihood of selecting a given position is proportional to the normalized activation values. This reflects their numerical magnitude, as shown in Equation 2.


(2) $$p_{i}=\frac{a_{i}}{\sum_{k\in R_{j}}a_{k}}$$

Where $p_{i}$ is the probability of each region j , $a_{i}$ denotes an element within the pooling region, and $R_{j}$ refers to the pooling region.

The location of the region l is sampled from a multinomial distribution according to the probabilities. Stochastic pooling is defined as Equation 3.


(3) $$Y_{j}=a_{l}\;\mathrm{where}\;l\sim P\left(p_{1},\;\ldots ,\;p_{\left|R_{j}\right|}\right)$$

Where $Y_{j}$ is the output of the pooling operation associated with the j-th feature map, and $a_{l}$ denotes the activation value of the pooling.

The specific process of stochastic pooling is as follows: The elements in the pooling region are normalized to obtain a probability matrix. Regions are then randomly selected based on these probabilities. The pooled value is the value of the selected region location.

**Figure 3:**
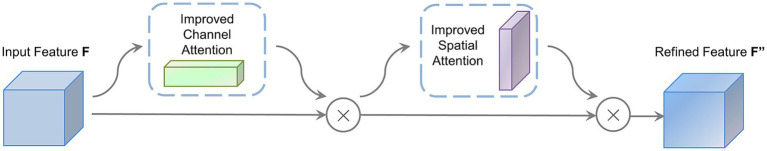
The proposed improved CBAM attention (IAT) block.

The improved CBAM attention block proposed in this paper is shown in Figure 3. The block receives the output feature map $F\in {\mathbb{R}}^{C\times H\times W}$ from the CV1 block. C , H , and W denote the channel count, height, and width of the feature map, respectively. This map serves as the input for the IAT block. First, the feature map passes through the channel attention block and produces a one-dimensional channel attention map $M_{c}\in {\mathbb{R}}^{C\times 1\times 1}$ . Second, $M_{c}$ is element-wise multiplied with F to emphasize important channels. This produces a modified feature map $F^{\prime }$ . Third, $F^{\prime }$ enters the spatial attention block. It generates a two-dimensional spatial attention map. $M_{S}\in {\mathbb{R}}^{C\times H\times W}$ . Finally, $M_{S}$ is element-wise multiplied with $F^{\prime }$ . This applies spatial attention weights to enhance spatial regions of the feature map. The resulting output is denoted as $F^{\prime \prime }$ .

**Figure 4:**
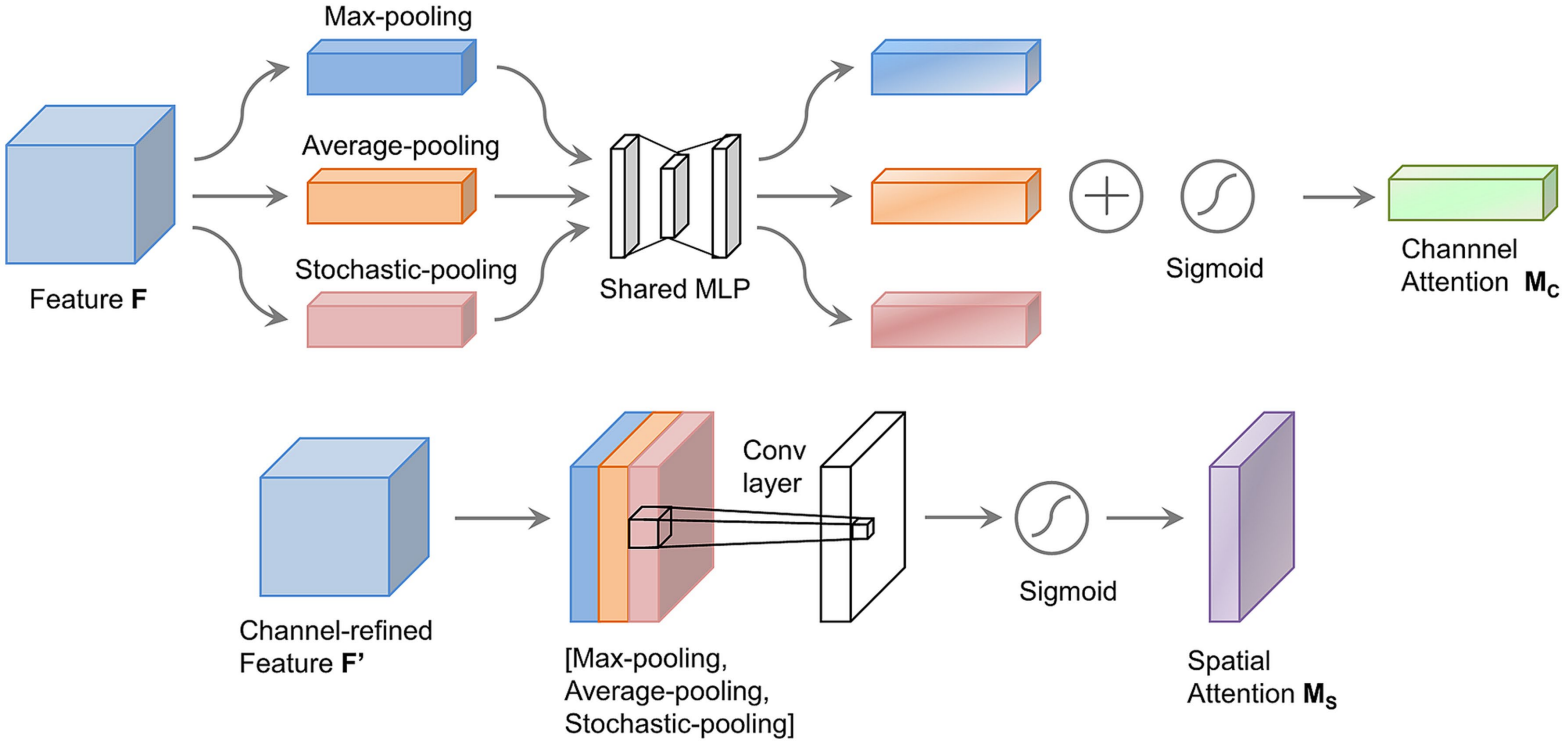
Improved channel attention block and improved spatial attention block.

The channel attention block within the IAT block integrates global information from the feature map. This provides a detailed representation of each channel, as shown in the upper half of Figure 4. First, three pooling strategies, average, max, and stochastic pooling, are applied to the input feature map $F\in {\mathbb{R}}^{C\times H\times W}$ . This broadens the spectrum of captured information. The resulting vectors are then reshaped to ${\mathbb{R}}^{C\times 1\times 1}$ dimensions. Second, these three channel description vectors are input into a multilayer perceptron (MLP) consisting of two FC layers. The MLP generates the attention weights for each channel. Rectified linear unit (ReLU) activation is incorporated within the MLP to enhance non-linearity. To reduce computational cost, the hidden layer’s size is set to ${\mathbb{R}}^{C/r\times 1\times 1}$ , with r as the reduction ratio. Finally, the aligned positions of the three feature vectors are summed, followed by the application of a sigmoid function to produce the channel attention map $M_{c}$ . The channel attention calculation is shown in Equation 4.


(4) $$\begin{array}{c} M_{C}\left(F\right)=\sigma \left(W_{1}\left(W_{0}\left(F_{avg}\right)\right)\right)+\sigma \left(W_{1}\left(W_{0}\left(F_{\text{max}}\right)\right)\right)\\ +\sigma \left(W_{1}\left(W_{0}\left(F_{sto}\right)\right)\right) \end{array}$$

Where σ is sigmoid activation function, while $F_{avg}$ , $F_{\text{max}}$ , and $F_{sto}$ denote the features derived from the channel attention block after applying average, max, and stochastic pooling, respectively. The MLP weights are represented by $W_{0}$ and $W_{1}$ , with a ReLU activation following $W_{0}$ . $W_{0}$ , and $W_{1}$ sequentially assign weights to the pooled features from each of the three pooling operations.

The spatial attention block in the IAT block focuses on the spatial aspects of the input feature map. It highlights important spatial locations and reduces less critical ones, as shown in the lower half of Figure 4. First, average, max, and stochastic pooling are applied across each channel of the feature map $F^{\prime }$ . Second, the resulting feature maps have identical dimensions. They are then concatenated and passed through a 2D convolution layer with a 7 × 7 kernel. Finally, a sigmoid function generates the spatial attention feature map $M_{S}$ . The computation for spatial attention is shown in Equation 5.


(5) $$M_{S}\left(F^{\prime }\right)=\sigma \left(f^{7\times 7}\left(\left[F_{avg}^{\prime };F_{\text{max}}^{\prime };F\prime_{sto}\right]\right)\right)$$

Where $f^{7\times 7}$ is the convolutional operation performed with a 7 × 7 kernel, while $F_{avg}^{\prime }$ , $F_{\text{max}}^{\prime }$ , and $F_{sto}^{\prime }$ denote the features derived from the spatial attention block after applying average, max, and stochastic pooling, respectively.

### 2.4 Temporal convolutional (TC) block

**Figure 5:**
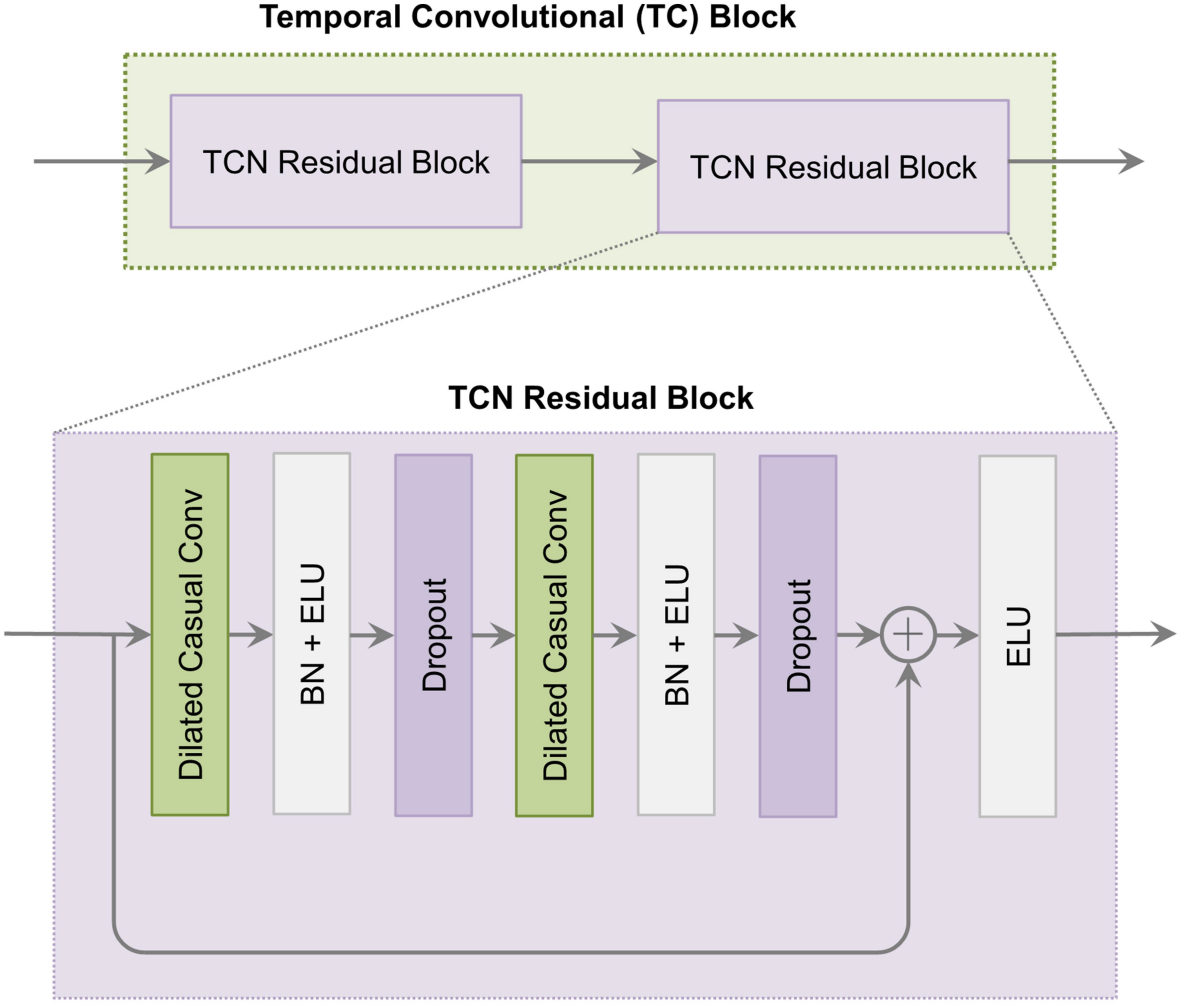
The architecture of the temporal convolutional (TC) block.

The TCN is particularly suited for processing sequential data. The TC block in this study follows the TCN architecture and consists of two residual blocks. Each residual block contains two dilated causal convolutional layers. After each convolutional layer, BN and ELU activation functions are applied. The architecture of the TC block is shown in Figure 5.

The TC block utilizes causal convolution. This ensures that model predictions at each step depend solely on prior inputs and are unaffected by future data. Additionally, it incorporates dilated convolution by introducing gaps in the convolution process. This allows the kernel to capture a broader temporal range. In residual blocks, residual connections alleviate vanishing and exploding gradient issues by summing input and output feature maps element-wise. With dilated convolution, the receptive field size (RFS) in the TC block increases exponentially with the dilation factor, as shown in Equation 6.


(6) $$RFS=1+2\left(K_{T}-1\right)\left(2^{L}-1\right)$$

Where $K_{T}$ is the kernel size, and L denotes the number of residual blocks.

Within the TC block, parameters $K_{T}=4$ and L=2 are applied, along with 32 filters. Given these settings, the RFS is 19. This allows the TCN to process sequences up to a maximum length of 19. Additionally, a parallel branch adjacent to the TC block outputs features from the IAT block. This setup enhances feature diversity by combining original features with those obtained after temporal convolution. Finally, the outputs from these two branches are concatenated with the CV2 block’s output. These are then fed into the FC layer with a softmax classifier. The hyperparameters used in the experiments are consistent across all subjects, as shown in Table 1.

**Table 1:** Hyperparameter settings used for all subjects.

Convolutional (CV) block		Improved CBAM attention (IAT) block	
CV1 Temporal filters (*F*_1_)	16	Reduction ratio	8
CV2 Temporal filters (*F*_3_)	32		
Kernel size (*K_C_*_1_)	32	Temporal Convolutional (TC) block	
Kernel size (*K_C_*_3_)	64	# of residual blocks ( L )	2
Depth multiplier ( D )	2	Kernal size (*K_T_*)	4
Average pool size	8	Fliters	32
Dropout rate	0.3	Dropout rate	0.3

## 3 Experimental and results

### 3.1 Dataset

The BCI IV-2a dataset (Brunner et al., 2008) serves as the benchmark for evaluating our proposed model. This publicly available MI-EEG dataset developed by Graz University of Technology is known. It contains a large number of artifacts, which increases the complexity of decoding MI tasks. It includes EEG recordings from nine healthy subjects, recorded with 22 electrodes following the standard 10–20 system at a sampling rate of 250 Hz. Signals are band-pass filtered between 0.5 and 100 Hz, with a 50 Hz notch filter applied to remove line noise. Each participant performs 576 trials of MI tasks, each lasting 4 s. Of these trials, 288 are used for training and the remaining 288 are reserved for evaluation. The MI tasks consist of four types: left-hand movement, right-hand movement, both feet movement, and tongue movement.

The BCI IV-2b dataset (Leeb et al., 2008) is also used to evaluate our proposed model. This dataset consists of EEG recordings from nine healthy subjects, captured using three electrodes at a 250 Hz sampling rate. Signals are band-pass filtered between 0.5 and 100 Hz, with a 50 Hz notch filter applied to remove line noise. Each participant completes five sessions: the first two without feedback, and the remaining three with smiley face feedback. Each session includes 120 trials of MI tasks, with each tasks lasting 4 s. The MI tasks consist of two types: left-hand movement and right-hand movement. Participants perform these tasks only through mental imagery, without any physical movement.

In this study, we use accuracy and kappa score to evaluate the performance of our proposed model. Accuracy provides a straightforward and intuitive measure, especially suitable for datasets with balanced class distributions. Kappa score accounts for biases due to imbalanced class distributions. It offers a more robust and impartial assessment of classifier performance compared to chance-level predictions. The equation for calculating accuracy (Equation 7) and kappa scores (Equation 8) are presented below:


(7) $$\mathrm{Accuracy}=\frac{1}{N}\overset{N}{\underset{i=1}{\sum }}\frac{TP_{i}}{I_{i}}$$

Where $TP_{i}$ is the true positives. It represents the number of samples correctly predicted within category i . $I_{i}$ denotes the total number of samples in category i . N refers to the total number of categories.


(8) $$\mathrm{Kappa}=\frac{1}{N}\overset{N}{\underset{a=1}{\sum }}\frac{P_{a}-P_{e}}{1-P_{e}}$$

Where $P_{a}$ is the actual percentage of agreement, $P_{e}$ denotes the expected percentage of agreement, and N refers to the total number of classes.

### 3.2 Other CNN-based models

Our proposed CIACNet model is compared with several CNN-based models. A description of each model is provided below:

1. EEGNet (Lawhern et al., 2018): A compact CNN model comprising a 2D convolutional layer, a depthwise convolutional layer, and a separable convolutional layer.
2. TCNet-Fusion (Musallam et al., 2021): This multi-branch model combines CNN and TCN architectures. The first block is based on EEGNet, while the second block splits the output into three branches, two remain unchanged, and the third connects to a TCN module. The third block concatenates the outputs from all branches, followed by classification with the softmax function.
3. ATCNet (Altaheri et al., 2022): Integrates attention mechanisms with TCN and CNN architectures. The attention block applies multi-head self-attention to identify key features. The TC block utilizes TCN to extract advanced temporal features.
4. TBTSCTnet (Chen X. et al., 2024): A multi-branch model incorporating Transformer with three branches that apply temporal and spatial convolutional filters of varying sizes. A Transformer encoder captures global dependencies within the spatiotemporal features extracted by the convolutional layers.

### 3.3 Training procedure

Experiments involving the proposed CIACNet model and the comparison models are conducted on an NVIDIA GTX 1650 8G, using TensorFlow 2.7 framework. Uniform training settings are applied across all models in this study. All models were trained using the Adam optimizer with a learning rate of 0.001 and a batch size of 64. Categorical cross-entropy loss was employed over 1,000 epochs. Model weights are saved only when there is an improvement in accuracy, which ensures the retention of the best-performing models. All models undergo validation based on supervised validation loss, with a reduced learning rate applied if validation loss stabilizes over consecutive epochs. To prevent overfitting, early stopping is used with the patience parameter set to 300 epochs. Each subject was trained 10 times. The best-performing trial among them was selected as the final result.

### 3.4 Comparison with other models

Tables 2, 3 offer a detailed overview of classification accuracy and kappa scores for CIACNet model and four other CNN-based models on the BCI IV-2a and IV-2b datasets. They also present the average values and standard deviations of the model parameters. The CIACNet model consistently outperforms reproduced EEGNet, TCNet-Fusion, ATCNet, and TBTSCTNet models. It demonstrates the best performance on both datasets. On the BCI IV-2a dataset, CIACNet model achieves an average accuracy of 85.15% and a kappa score of 0.80. It exceeds the advanced ATCNet model by 1.97% and outperforms the other models by at least 3.52%. With an accuracy standard deviation of 8.41%, CIACNet model also demonstrates the highest stability in accuracy across all subjects. Additionally, on the BCI IV-2b dataset, CIACNet model achieves an average accuracy exceeding 90%. This demonstrates its significant advantage in classifying MI tasks for left and right hand movements. To show the role of our model in reducing training time costs, we add the average training time (in minutes) for each model when classifying all subjects in the last row of Tables 2, 3. On the BCI IV-2a dataset, EEGNet and TCNet-Fusion model require the shortest training times, but they also achieve the lowest classification accuracy. Compared with the remaining two models, our CIACNet achieves the shortest training time, averaging only 12.01 min. A similar conclusion is drawn for the BCI IV-2b dataset, where our model shows significantly shorter training times than the ATCNet model.

**Table 2:** Comparison of classification accuracy (%) and kappa between the proposed model and other reproduced models on the BCI IV-2a dataset.

Subject	EEGNet		TCNet-Fusion		ATCNet		TBTSCTNet		CIACNet (ours)	
	Acc	Kappa	Acc	Kappa	Acc	Kappa	Acc	Kappa	Acc	Kappa
A01	85.76	0.81	85.42	0.81	86.11	0.81	87.80	0.84	90.63	0.88
A02	60.42	0.47	60.76	0.48	69.44	0.59	61.46	0.49	75.69	0.68
A03	92.71	0.90	89.58	0.86	95.14	0.94	93.40	0.91	96.53	0.95
A04	63.54	0.47	63.19	0.51	80.21	0.74	78.13	0.71	80.56	0.74
A05	73.61	0.65	73.26	0.64	81.25	0.75	79.17	0.72	82.64	0.77
A06	60.76	0.48	62.50	0.50	69.10	0.59	65.28	0.54	70.49	0.61
A07	89.93	0.87	90.28	0.87	90.28	0.87	92.01	0.89	92.01	0.89
A08	81.25	0.75	84.03	0.79	87.85	0.84	87.85	0.84	88.89	0.85
A09	82.99	0.77	85.76	0.81	89.24	0.86	89.58	0.86	88.89	0.85
Mean	76.77	0.69	77.20	0.70	83.18	0.78	81.63	0.76	**85.15**	**0.80**
Std	12.63	0.17	12.30	0.16	9.08	0.12	11.61	0.15	8.41	0.11
Time	4.00		7.92		13.70		15.14		12.01	

The bold font highlights the best results among different models.

**Table 3:** Comparison of classification accuracy (%) and kappa between the proposed model and other reproduced models on the BCI IV-2b dataset.

Subject	EEGNet		TCNet-Fusion		ATCNet		TBTSCTNet		CIACNet (ours)	
	Acc	Kappa	Acc	Kappa	Acc	Kappa	Acc	Kappa	Acc	Kappa
B01	77.81	0.56	76.56	0.53	74.06	0.48	76.25	0.53	79.06	0.58
B02	72.50	0.45	74.64	0.49	72.50	0.45	75.36	0.51	76.43	0.53
B03	88.75	0.78	88.44	0.77	88.75	0.78	87.81	0.76	90.00	0.80
B04	97.19	0.94	98.13	0.96	98.13	0.96	98.13	0.96	98.75	0.98
B05	95.00	0.90	96.57	0.93	95.94	0.92	96.25	0.93	98.44	0.97
B06	89.69	0.79	86.25	0.73	88.13	0.76	87.50	0.75	90.31	0.81
B07	89.38	0.79	88.13	0.76	93.75	0.88	89.69	0.79	93.75	0.88
B08	91.56	0.83	95.00	0.90	94.69	0.89	95.63	0.91	95.94	0.92
B09	85.60	0.71	86.88	0.74	89.06	0.78	87.81	0.76	87.81	0.76
Mean	87.50	0.75	87.84	0.76	88.33	0.77	88.27	0.77	**90.05**	**0.80**
Std	7.90	0.16	8.21	0.16	9.21	0.18	8.13	0.16	7.95	0.16
Time	2.23		3.11		12.40		9.76		6.72	

The bold font highlights the best results among different models.

**Figure 6:**
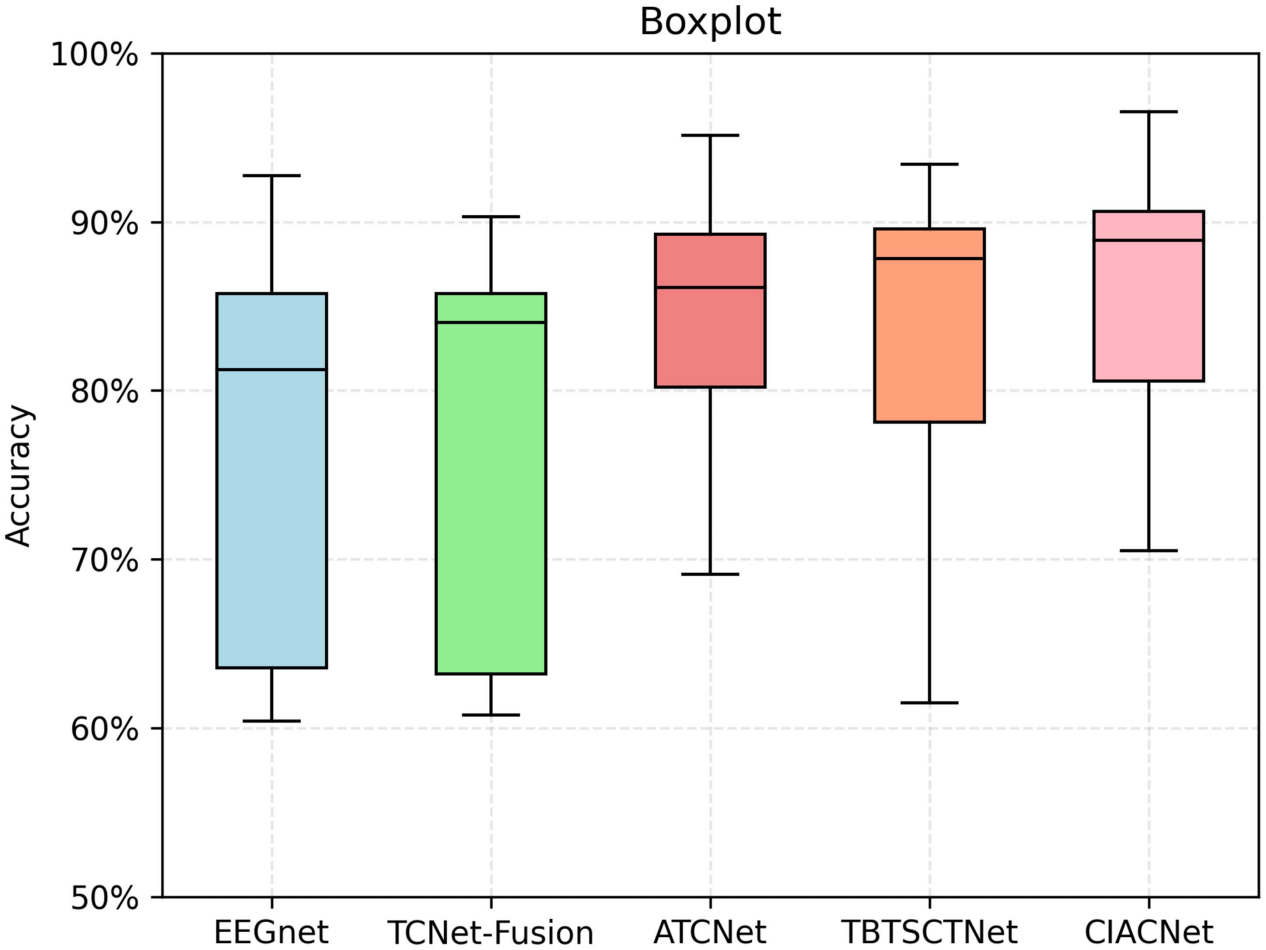
Boxplot of classification accuracies for different models on the BCI IV-2a dataset.

Boxplots are used to visually assess the performance of the CIACNet model and other models. They show the distribution of accuracy across each model. Figure 6 displays the boxplot for the classification results of the five models on the BCI IV-2a dataset. In the boxplot, the x-axis represents the model names and the y-axis shows accuracy. The horizontal lines within each boxplot indicate the median accuracy, while the whiskers denote the minimum and maximum values across all subjects. The edges of each box correspond to the upper and lower quartiles. The figure clearly shows that the CIACNet model exceeds the other models in terms of median accuracy, as well as in maximum, minimum, and quartile values, across all subjects.

**Figure 7:**
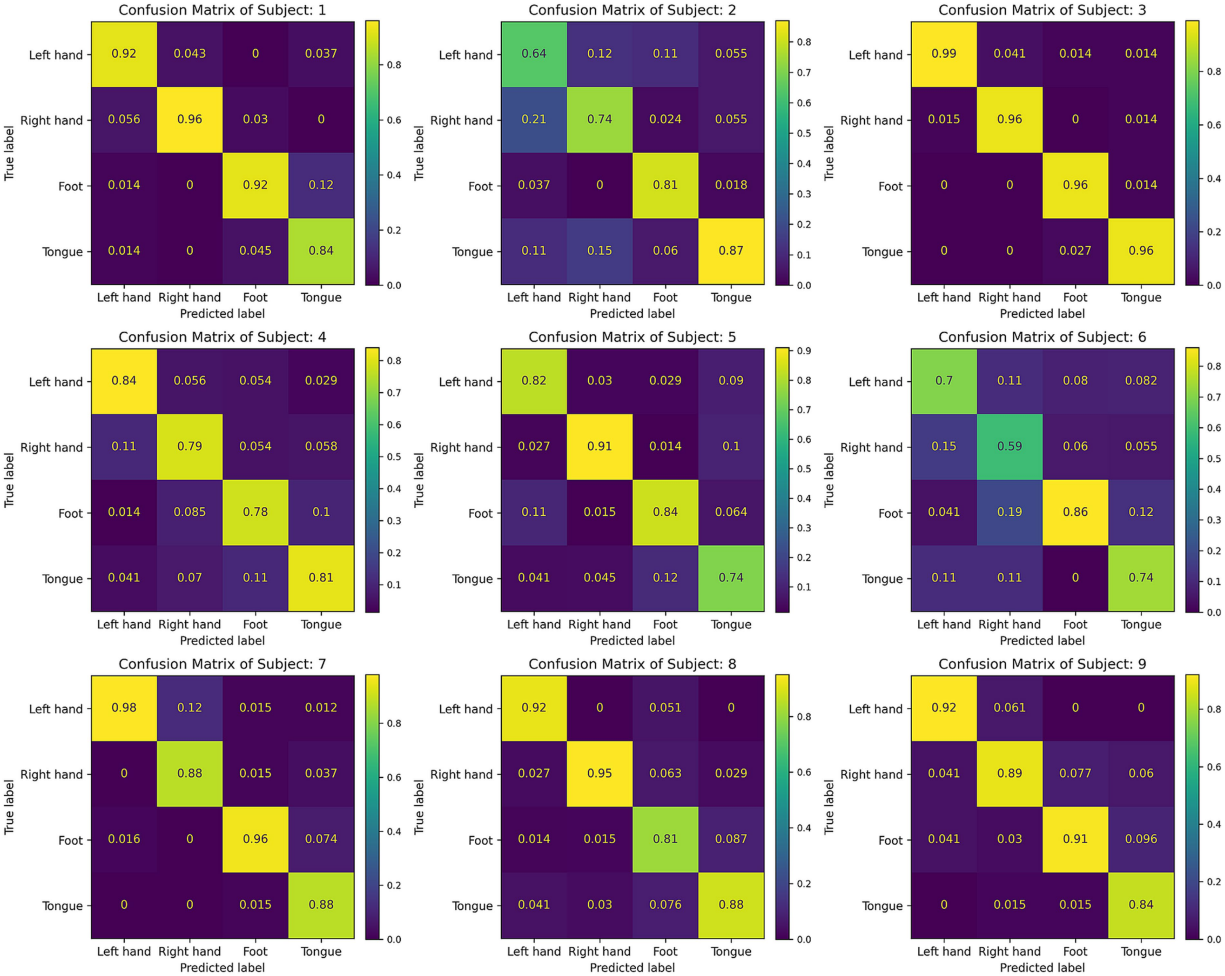
Confusion matrix for all 9 subjects of the BCI IV-2a dataset.

**Figure 8:**
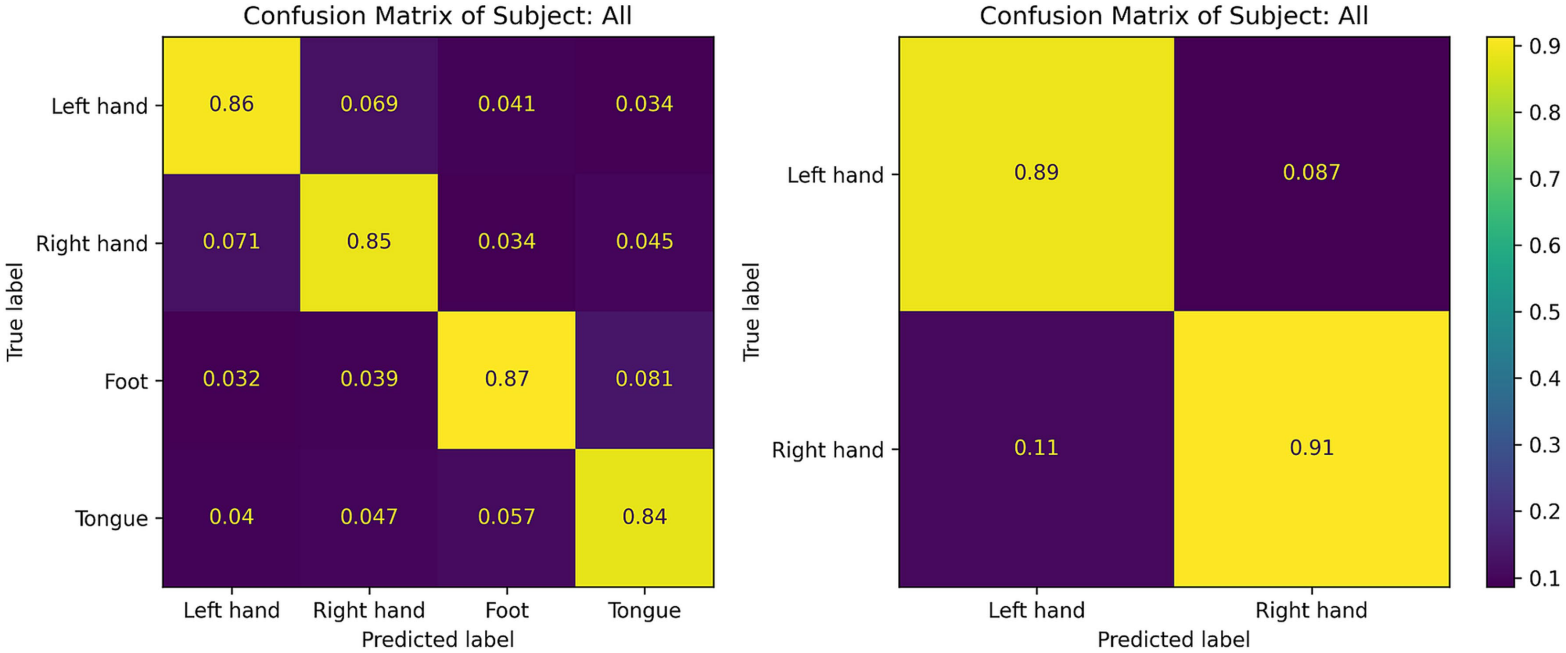
The subject average confusion matrix of the CIACNet model on the BCI IV-2a and BCI IV-2b datasets.

Figure 7 displays the confusion matrices for the CIACNet model’s classification results on the BCI IV-2a dataset. These figures show the model’s strong performance across all subjects. In Figure 7, subjects S1, S3, and S7 achieve classification accuracies exceeding 90%. Figure 8 shows the mean confusion matrices across all subjects for BCI IV-2a and BCI IV-2b datasets as classified by the CIACNet model. The left panel of Figure 8 shows average accuracies of 86, 85, 87, and 84% for the left hand, right hand, both feet, and tongue tasks, respectively. The right panel shows average accuracies of 89% for the left hand and 91% for the right hand. These results suggest that the CIACNet model consistently achieves balanced and high classification accuracy on both datasets. This demonstrates its stability and precision in decoding MI-EEG signals. It also highlights the model’s potential for real-world applications.

Table 4 presents the classification results of the CIACNet model on the BCI IV-2a and BCI IV-2b datasets. It also provides a comparative analysis with other state-of-the-art (SOTA) models. The table details the main methodologies used by these models, which include multi-branch multi-scale and attention mechanisms. The CIACNet model outperforms these SOTA models in terms of both accuracy and kappa scores for the four-class and two-class classification tasks. This indicates the high performance of our model in classification tasks.

**Table 4:** Classification performance of different models based on the BCI IV-2a and BCI IV-2b datasets.

BCI IV-2a			BCI IV-2b		
Method	Acc	Kappa	Method	Acc	Kappa
DSCNN: multi-branch CNN (Ma et al., 2022)	84.64	0.79	RSMM: robust support matrix machine (Zheng et al., 2018)	77.97	0.60
self-attention and CSP-based CNN (Zhang et al., 2023)	79.28	N/A	DAFS (Phunruangsakao et al., 2022)	84.63	0.73
IFNet: interactive frequency CNN (Wang et al., 2023)	78.21	N/A	Global Adaptive Transformer (Song et al., 2023)	84.44	0.69
EEG Conformer: CNN and self-attention (Song et al., 2022)	78.66	0.72	EEG Conformer: CNN and self-attention (Song et al., 2022)	84.63	0.69
MBMANet: multi-branch CNN and attention (Deng et al., 2024)	83.18	0.78	EEGNet Fusion V2: five-branch CNN (Chowdhury et al., 2023)	84.10	N/A
FCNNA: dual-branch CNN and CBAM (Khabti et al., 2024)	83.78	0.78	MT-MBCNN: multi-task multi-branch CNN (Cai et al., 2024)	81.40	N/A
CIACNet: CNN, improved CBAM, and TCN (ours)	85.15	0.80	CIACNet: CNN, improved CBAM, and TCN (ours)	90.05	0.80

### 3.5 Ablation experiment

We conduct ablation studies to evaluate the contribution of each block within the CIACNet model. Table 5 shows the effects of removing one or more blocks on the CIACNet model’s performance. It presents the average accuracy and kappa scores for classification tasks on both the BCI IV-2a and BCI IV-2b datasets. For the BCI IV-2a dataset, the CV2 and IAT blocks improve average accuracy by 2.52 and 2.86%, respectively. Removing the TC block leads to a 3.21% decrease in average accuracy. This suggests that CBAM may not be suitable when positioned only in the final CNN layer. For the BCI IV-2b dataset, removing the TC block causes a more significant reduction in accuracy than removing the CV2, IAT, and TC blocks together. This highlights the importance of the TC block for this relatively simpler dataset. The CIACNet model utilizes a hybrid approach, integrating CBAM elements into both intermediate and final layers. This enhances its ability to focus attention across different feature levels. The ablation study in Table 5 shows that removing one or more blocks results in a decline in the overall performance of the CIACNet model. Therefore, each block within the model makes a significant contribution to its overall effectiveness.

**Table 5:** The contribution of each block in CIACNet model was evaluated using the BCI IV-2a and BCI IV-2b datasets.

Removed block	BCI IV-2a		BCI IV-2b	
	Acc	Kappa	Acc	Kappa
None (CIACNet)	85.15	0.80	90.05	0.80
CV2	82.63	0.77	89.09	0.78
IAT	82.29	0.76	88.81	0.78
CV2 + IAT	80.24	0.74	87.78	0.76
TC	81.94	0.76	87.19	0.74
CV2 + TC	81.90	0.76	87.05	0.74
IAT + TC	81.20	0.75	88.13	0.76
CV2 + IAT + TC	79.20	0.72	87.61	0.75

Moving forward, we assess the impact of modifying the attention mechanisms within the IAT block on the CIACNet model’s performance. Table 6 presents the accuracy and kappa scores after substituting the improved CBAM in the IAT block with standard CBAM, SE, and MSA, respectively. The first row in the table represents the original setup, which uses the improved CBAM. Results in Table 6 indicate that the improved CBAM achieves the highest accuracy and kappa scores. Across both datasets, the accuracy with the improved CBAM exceeds other attention mechanisms by at least 0.77 and 0.35%, respectively. Additionally, we compare the performance of the improved CBAM and the standard CBAM on EEG data with high noise levels. On the BCI IV-2a dataset, we focus on high-noise subjects 2 and 6. Using the model with the improved CBAM, the accuracies for subjects 2 and 6 are 75.69 and 70.49%, respectively. In contrast, the model with the standard CBAM achieves accuracies of 73.96 and 68.40% for these subjects. The comparison results indicate that the improved CBAM outperforms the standard CBAM in handling high-noise EEG data. Finally, as shown in Table 6, the improved CBAM shows its advantages in classification tasks for both large datasets (BCI IV-2a) and small datasets (BCI IV-2b). Thus, our proposed IAT block significantly improves the model’s ability to extract key features, thereby enhancing its decoding performance.

**Table 6:** The impact of changing the attention mechanism on CIACNet model performance.

Changed block	BCI IV-2a		BCI IV-2b	
	Acc	Kappa	Acc	Kappa
None (CIACNet)	85.15	0.80	90.05	0.80
CBAM	84.38	0.79	89.70	0.79
SE	84.03	0.79	89.59	0.79
MSA	83.68	0.78	88.74	0.78

### 3.6 Visual analyses

**Figure 9:**
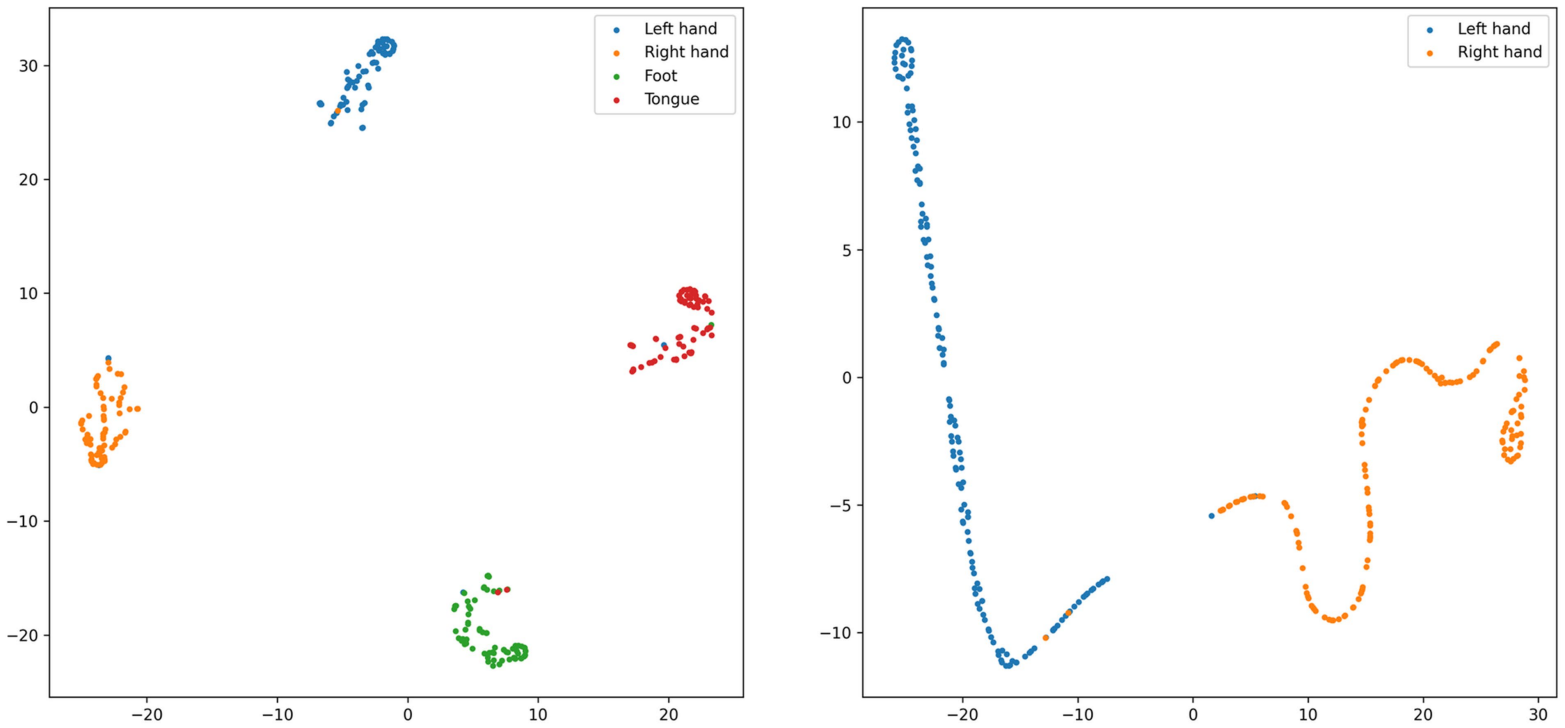
t-SNE visualization of subject 3 from the BCI IV-2a dataset and subject 4 from the BCI IV-2b dataset.

Figure 9 presents a visualization analysis of the CIACNet model’s classification results on the BCI IV-2a and BCI IV-2b datasets. The analysis uses t-distributed stochastic neighbor embedding (t-SNE). The t-SNE technique projects high-dimensional samples into a low-dimensional space, preserving the relative proximity of similar samples. All feature map dimensions have been reduced to two dimensions using t-SNE for this analysis. In Figure 9, points in four distinct colors represent classification results for the left hand, right hand, both feet, and tongue tasks. The x and y axes correspond to these two projected dimensions. The analysis includes subject 3 from the BCI IV-2a dataset and subject 4 from the BCI IV-2b dataset exhibiting best performance. For subject 3, classification accuracy is 98.6% (71/72) for the left hand and 95.8% (69/72) for the right hand, both feet, and tongue tasks. Subject 4 achieves 98.6% (158/160) accuracy for both left and right hand tasks. These results demonstrate the model’s high classification proficiency and its capability to delineate clear cluster boundaries.

## 4 Discussion

In this study, we propose a CIACNet model that integrates dual-branch CNN, improved CBAM, TCN, and multi-level feature concatenation to address the challenges in motor imagery EEG classification.

CIACNet is a lightweight EEG decoding model that requires only minimal preprocessing, limited to standardizing the input data. The CV block uses two branches to process MI-EEG signals in parallel. This allows extracting rich temporal features. The IAT block utilizes an improved CBAM to effectively focus on the key features of the EEG signals. The TC block employs TCN to capture high-level temporal features from sequential data. The multi-level feature concatenation enhances the model’s ability to represent complex features.

In the experiments, the CIACNet model achieves the best results on both the BCI IV-2a and BCI IV-2b datasets. Specifically, the model achieves an accuracy of 85.15% and a kappa score of 0.80 on the BCI IV-2a dataset. On the BCI IV-2b dataset, it achieves an accuracy of 90.05% and a kappa score of 0.80. Compared to the four other models included in the comparison, CIACNet demonstrates superior performance. The classification of MI-EEG signals remains a challenging task in current research. Existing results from SOTA models show limited improvements in MI-EEG signal classification algorithms. For instance, on the BCI IV-2a dataset, the EEG Conformer model proposed by Song et al. (2022) achieves an accuracy of 78.66%, representing a 2.46% improvement over previous models. The simplicity of the model limits its ability to achieve better classification performance. The MBMANet model proposed by Deng et al. (2024) achieves an accuracy of 83.18%, marking a 3.43% increase. On the BCI IV-2b dataset, the EEGNet Fusion V2 model proposed by Chowdhury et al. (2023) achieves an accuracy of 84.10%. This model focuses solely on the number of CNN branches and lacks additional blocks to further enhance performance. The proposed CIACNet model outperforms these studies on both datasets.

Ablation experiment confirms the necessity of each block within the CIACNet model for improving overall performance. When the CV2 block, IAT block, or TC block is removed from the model, its performance decreases. The performance decline becomes more pronounced when multiple blocks are removed. Additionally, we observe that the IAT block performs more effectively when placed in the middle of the model. By modifying the attention mechanism, we observe that our improved CBAM results in an increase of at least 0.77 and 0.35% in accuracy across both datasets compared to other attention mechanisms. This result verifies that the improved CBAM is highly effective at enhancing the model’s ability to extract key information. Our improved CBAM modifies the pooling approach of the standard CBAM. We add stochastic pooling to standard CBAM. Stochastic pooling assigns probabilities to feature map elements based on their numerical values. This increases the diversity of pooling, thereby enhancing the model’s ability to focus on key features.

We also present the confusion matrices and t-SNE images. In Figure 7, most subjects show good classification performance. However, subject 2 and subject 6 exhibit lower classification accuracy for left and right hand tasks. This indicates the presence of more artifacts in their EEG signals. In Table 2, the proposed model still significantly outperforms the other four models in terms of classification performance for subject 2. This demonstrates that our model maintains better performance when handling EEG data with more artifacts. The model captures key features and performs multi-level feature concatenation, reducing the impact of artifacts. The t-SNE images show that, for subjects with high classification accuracy, the proposed model effectively delineates cluster boundaries.

The proposed model in this study can be applied to the development of MI-BCI systems. By integrating with other devices, MI-BCI systems can achieve high accuracy. When utilized for upper limb rehabilitation in stroke patients, a decoding algorithm with high accuracy can enhance the precision of the feedback mechanism, thereby improving rehabilitation outcomes. Despite the advancements in MI-EEG signal decoding achieved in this study, several challenges remain. The proposed model is trained and validated individually for each subject, limiting its ability to effectively utilize information from other subjects. Future work will focus on incorporating algorithms such as transfer learning to enhance the generalizability of the models. We also plan to develop adaptive attention mechanisms. These mechanisms will automatically adjust attention allocation based on the varying needs of individuals and tasks. This enhances the versatility of decoding algorithms. We are confident that these efforts will drive further progress in BCI technology, bringing us closer to broader adoption of BCI applications.

## 5 Conclusion

This study proposes a composite improved attention convolutional network (CIACNet) for MI-EEG signals classification. The CIACNet model consists of three main components: the CV, IAT, and TC blocks. The experimental results from the BCI IV-2a and BCI IV-2b datasets shows that our model achieves accuracies of 85.15 and 90.05%, respectively. It outperforms the four other models under comparison. This demonstrates that our CIACNet model achieves high classification accuracy. It does not require artifact removal and has a short training time, thereby reducing the time cost in MI-BCI systems.

## Data Availability

The datasets presented in this study can be found in online repositories. The names of the repository/repositories and accession number(s) can be found in the article/supplementary material.
